# Benefit or Problem: Exploration of How Response Options Affect Self-Reported Behaviors and Interests in Autistic Adults

**DOI:** 10.3390/healthcare12090911

**Published:** 2024-04-27

**Authors:** Hyein Lee, Nikita Jadav, Ellen Wilkinson, Vanessa H. Bal

**Affiliations:** 1 Department of Clinical Psychology, Graduate School of Applied and Professional Psychology, Rutgers University, Piscataway, NJ 08854, USA; 2 Rutgers Robert Wood Johnson Medical School, New Brunswick, NJ 08901, USA; nikita.jadav@rutgers.edu; 3 Department of Psychology, Rutgers University, Piscataway, NJ 08854, USA; ellen.wilkinson@rutgers.edu; 4 Department of Applied Psychology, Graduate School of Applied and Professional Psychology, Rutgers University, Piscataway, NJ 08854, USA

**Keywords:** autism, restricted, repetitive behavior, neurodiversity

## Abstract

Assessment of restricted, repetitive behaviors (RRB) in autism evaluations often assumes that these behaviors negatively impact the individual. Qualitative studies of first-person accounts indicate the negative impact of the stigma associated with RRBs but also provide insights into the positive aspects. The current study explores how framing response options as negative (i.e., level of problem associated with occurrence) or positive (i.e., level of benefit associated with occurrence) affects RRB self-reports in autistic adults. Sixty-six autistic adults aged 18–59 filled out the Repetitive Behavior Scale-Revised (RBS-R) and a modified RBS-R+, assessing problems and benefits of reported behaviors, respectively. There was a moderate to strong correlation between the forms, each assessing problems and benefits in terms of the number of behaviors endorsed (r = 0.746) and the levels of benefits and problems (r = 0.637). Autistic adults reported a higher number of RRBs in the form that assessed problems, but the number of behaviors was comparable between the forms when counting in the response option of the occurrence of behavior without having a benefit. Despite some variability in the level of problems and the benefits across the subdomains of RRB, autistic adults largely rated comparable levels of associated benefits and problems, highlighting the complexity of RRBs as having both positive and negative impacts. Future screening and diagnostic tools for adults should aim to assess both positive and negative aspects of autistic features to afford a more nuanced understanding of individual experiences while still yielding diagnostically relevant information. Qualitative studies are needed to better understand the complex experiences associated with these behaviors; however, it may be important to ensure that options for endorsement of behaviors without a specific benefit are also needed to ensure some behaviors (e.g., self-injurious behaviors) are not missed.

## 1. Introduction

Restricted, repetitive patterns of behaviors (RRB) are a group of heterogeneous behaviors that have been included as core features of autism since Kanner’s [1] early descriptions. Consistent with the focus of DSM-5 diagnostic criteria, assessment of RRBs often focuses on identifying the presence of these behaviors and establishing ways in which these behaviors interfere with daily, social, occupational, or other areas of functioning. Recently, however, first-person accounts of these behaviors have afforded a more strengths-based view, providing insight into the potential benefits and adaptive aspects of those behaviors that could serve as advantages [2,3,4]. The present study sought to explore how framing response options as inherently negative (impairing) or positive (beneficial) may affect the self-reports of these behaviors in autistic adults.

The literature highlights several advantages to behaviors that fall in the RRB category. Self-reports from children and adults indicate that mannerisms, such as stimming, may be used to regulate emotional responses to external and internal stressors [3,4,5]. In addition, passionate interests (a term suggested by Bottema-Beutel [6] as an alternative to “special interest”, commonly used to refer to the diagnostic criterion of restricted, fixated interests) can contribute to an increase in positive emotions, such as enthusiasm, pride, and happiness, and enhance self-confidence and promote positive self-image, which could, in turn, enhance communication skill and social connection [7]. Passions can also be effectively incorporated into children’s educational programming to increase interest and motivation when teaching academic, adaptive, play, and social skills [8,9]. In a study of autistic youth and their parents, linking specific interests to ideas about the future profession helped them to develop clear career plans and goals [7]. In a systematic review, themes identified as positive contributors to autistic adults’ work performance included several behaviors that are often categorized as RRBs, including attention to detail, tolerance of repetitive tasks, and special interests [10].

Studies exploring autistic experiences suggested that features or behaviors falling in different subdomains of RRBs may have different effects. For example, sensory sensitivity was reported to have negative physical, emotional, and cognitive effects [5]. Self-injurious behaviors were also viewed as having a negative impact due to the resulting physical harm [11]. Other behaviors, such as stimming, were associated with both positive and negative experiences. While emotional expression and cognitive self-regulation were benefits associated with stimming, adults felt they had to suppress their stims due to stigma and social pressure [5]. Similarly, while engaging in interests evoked positive emotions, absorption or difficulty leaving something incomplete had a negative impact on time management, sleep, or other activities [11].

RRBs have been assessed using a variety of methods, including observations of behaviors by trained researchers or clinicians, as well as self- and informant-completed questionnaires or interviews. The Repetitive Behaviors Scale-Revised (RBS-R) [12] is a questionnaire widely used in research and clinical settings. The RBS-R has 43 items organized into six conceptually-driven categories: Stereotypic Behavior; Self-Injurious Behavior; Compulsive Behavior; Ritualistic Behavior; Sameness Behavior; and Restricted Behavior. Each behavior is rated on a scale from 0 (behavior does not occur) to 3, with scores of 1–3 indicating that the behavior occurs and is a mild, moderate, or severe problem self-report version of the adults [13,14].

Many studies report a tendency for males to score higher than females on quantitative measures of RRBs, including the RBS-R [15]. More recently, there has been a suggestion that behaviors most common in females may not be represented on the instruments used to evaluate RRBs, thereby contributing to the misconception that females have fewer RRBs [16,17]. This certainly could contribute to differences identified on the RBS-R. For example, the RBS-R has only one item related to sensory features, which is an area thought to be important in increasing diagnostic sensitivity in females [16]. Also, behavioral examples for some items on the RBS-R may be perceived as male-oriented [18]. Cross-sectional research suggests that fewer behaviors are endorsed on the RBS-R in adults compared to children [13], and longitudinal studies have suggested a tendency for RRBs to decrease across childhood [19]. This may also reflect both a reduction in certain features and a lack of items capturing adult presentation. An additional measurement component that has been less consistently explored, however, is the valence of response options (i.e., if the behavior is rated negatively/as being a problem vs. positively/as having some benefit). It has long been widely acknowledged how questions and response options affect self-report [20]. Self-evaluations of positive or negative impact have not been systematically evaluated in quantitative measurement of RRBs but could be another source of gender and/or age-related differences, therefore warranting further exploration.

Because the RBS-R response choices only allow the individuals to rate some degree of problem for behaviors that are present, it is not clear how autistic individuals would rate behaviors that are perceived to be present without a negative impact or behaviors that are perceived to have a benefit [14]. Focusing on negative impacts could result in underreporting behaviors that are important to inform diagnostic conceptualization. Restricting focus on problematic aspects of the behavior also could inadvertently reinforce misconceptions that all RRBs are problematic and contribute to adults feeling the need to mask or camouflage even those behaviors they view as bringing benefit to their lives [14,21]. This could have negative effects on autistic individuals’ self-image and mental health either directly, by resulting in feelings of shame, or indirectly, by keeping them from using effective coping strategies [4].

While focusing on frequency without qualifiers may ensure the capture of behaviors, ratings of positive or negative impact can also help clinicians and researchers understand individual experiences and better support autistic adults. Self-ratings of behaviors as beneficial may pinpoint areas of strength, whereas behaviors indicated to only be problematic may signify an area the person desires to support. On the other hand, ratings as both problematic and beneficial could indicate further consideration of causes or factors driving opposing experiences that critically inform appropriate intervention [16].

The objective of the current study was to gain insight into how response options affect self-report of RRBs. To do this, the current study utilized two versions of the RBS-R: the standard RBS-R, which assesses the presence and perceived level of problem of each behavior, and a modified RBS-R (RBS-R+), which reworded response options to capture the occurrence and level of perceived benefit associated with each behavior. Using these two forms, we had three aims: (1) explore whether the framing of behaviors as problem versus benefit affected the number of self-reported behaviors; (2) compare whether broad domains of RRBs were perceived as having a greater degree of problems or benefits; and (3) investigate if perceptions of behaviors (i.e., level of problem and benefit) varied by age or gender identity. As this is the first study to directly compare the same behaviors rated separately as either problematic or beneficial, these aims were considered exploratory with no specific hypotheses other than an expectation that autistic adults would report both problems and benefits associated with their RRBs. Notably, the RBS-R was selected due to its negatively oriented response options and many items designed to capture different types of RRBs, allowing for the modification to evaluate the effect of positively oriented response options on a range of behaviors. The aim of this study, however, was not to specifically inform modification to the RBS-R. Rather, the information about response options gained from this study is hoped to broadly inform the development or revision of measures to provide a more nuanced and clinically sensitive view of RRBs that acknowledges both potential benefits and problems associated with these behaviors.

## 2. Methods

### 2.1. Participants

Participants were 66 adults (40 females, 15 males, 11 identifying as nonbinary) with Autism Spectrum Disorder (ASD) diagnoses ranging in age from 18 to 59 years old (M = 28.24, SD = 9.34; Table 1). Self-reported age of autism diagnosis ranged from 2 to 56 years old (M = 20.61, SD = 12.24). To be eligible for participation, adults needed to (1) be 18 years or older, (2) have the ability to provide self-reports of their behaviors, interests, and experiences in English, and (3) self-report having a previous diagnosis of ASD (including DSM-5 ASD or DSM-IV Autistic Disorder, Asperger’s Syndrome, Pervasive Developmental Disorder, Not Otherwise Specified) provided by a healthcare professional. There was no further step to confirm the diagnosis; however, on the Social Responsiveness Scale 2 (Constantino and Gruber 2012), 83.3% of the participants fell above the T-score cut-off of 60 (*N* = 55).

### 2.2. Procedures

Prior to the survey being distributed, an autistic consultant reviewed questionnaire wording and response options and provided feedback. Participants were recruited online from ASD research support organizations, ASD advocacy and support groups, college autism support programs, and social media. Of 114 adults who completed a screening form linked from the flyer, 102 independent adults (*n* = 94) or their guardians (*n* = 8) (89% response rate) completed the study consent. Following the consent, participants were presented with a battery of questionnaires; the demographic form, SRS-2, and two RBS-R versions were administered at the beginning of the battery. Of those who consented, 66 completed the questionnaires (65% response rate). Participants were entered into a lottery for $25 gift cards (1 in 10). A subset of participants participated in interviews to gain insight into their RRBs (qualitative findings will be reported separately). All study procedures were approved by the Rutgers University Institutional Review Board (IRB; approval # Pro2020001507).

### 2.3. Measures

Repetitive Behavior Scale-Revised (RBS-R) [12]. The RBS-R is a self- or informant-report questionnaire that consists of 43 items that assess the presence of RRB and its severity within six subdomains (i.e., Stereotyped Behavior, Self-Injurious Behavior, Compulsive Behavior, Ritualistic Behavior, Sameness Behavior, and Restricted Behavior). A self-report version has emerging support for its reliability and validity in adults [14].

In the current study, two self-report versions of the RBS-R were administered. The RBS-R was the original questionnaire [12] with a minor wording modification (the phrase “apparently purposeless” was removed from the definition of Stereotyped Behavior at the suggestion of an autistic consultant). Per the original RBS-R design, participants rated items on a 4-point scale, where (0) reflects “behavior does not occur” and scores from (1) to (3) reflect that the behavior occurs and is a mild, moderate, or severe problem, respectively. Cronbach’s alpha was 0.944 for the overall total, and subdomains ranged from 0.747 (Stereotyped) to 0.868 (Sameness). The *RBS-R+* was identical to the original RBS-R, except for a modification in the response options. On the RBS-R+, (0) reflects that “behavior does not occur”, and scores from (1) to (3) reflect that the behavior occurs and has a mild, moderate, or significant benefit, respectively. The RBS-R+ additionally included an option of “behavior does occur and does not have a benefit”. Cronbach’s alpha was 0.966 for the overall RBS-R+ total, and subdomains ranged from 0.722 (Restricted Behavior) to 0.915 (Sameness). In the current study, the order of the RBS-R and the RBS-R+ presentation was counterbalanced (*N* = 32, 48.5% completed RBS-R first). The order of questionnaire presentation did not differ by gender identity (*p* = 0.88), race (*p* = 0.43), education (*p* = 0.32), and employment status (*p* = 0.43).

### 2.4. Analysis

#### Scoring of the RBS-R and the RBS-R+

*The number of behaviors endorsed.* To capture the number of behaviors endorsed by each participant, endorsements from 1 to 3 on both forms were rescored as 1 (capturing the presence of a particular behavior), and scores of 0 remained 0 (indicating absence of behavior). To allow for the comparison of behaviors endorsed with benefit to those endorsed with the problem, the number of items endorsed as “behavior does occur and does not have a benefit” on the RBS-R+ were tracked but not included in the RBS-R+ endorsement total.

*Level of impact endorsed.* RBS-R impact scores were derived by adding up the scores for each item to reflect a cumulative level of problems endorsed for each domain and overall total. RBS-R+ impact total and domain scores were derived using the same method to capture the level of perceived benefit associated with the RRBs. Items endorsed as “behavior does occur and does not have a benefit” on the RBS-R+ were scored as 0 to allow for a comparison of RBS-R scores reflecting the level of problem and RBS-R+ scores reflecting the level of benefit.

### 2.5. Statistical Analyses

Pearson correlations and paired sample *t*-tests were conducted to examine the relationship between the two forms (i.e., RBS-R; RBS-R+) and to explore how response options affected the number of behaviors and level of impact endorsed. Paired sample *t*-tests were also used to examine order effects. Finally, the correlations and one-way ANOVA with post-hoc Tukey tests were used to explore whether RBS-R or RBS-R+ endorsements were associated with age or gender identity. Order was evenly counterbalanced across gender identity; therefore, this factor was not controlled for in comparisons.

## 3. Results

### 3.1. Comparison of RBS-R and RBS-R+

As shown in Table 2, a strong positive correlation was found between the number of behaviors and the level of impact endorsed on the two RBS-Rs (behaviors endorsed: *r* = 0.746; level of impact: *r* = 0.637). Subdomain scores from the two measures were also positively correlated (*r* = 0.568 to 0.778 for behaviors endorsed and *r* = 0.516 to 0.643 for level of impact).

As shown in Table 3, fewer behaviors were endorsed as present with some benefit on the RBS-R+ than behaviors endorsed as present with some problem on the RBS-R (t(65) = −3.31, *p* = 0.002). Notably, many items were rated as present but did not have a benefit on the RBS-R+ (*M* = 4.50, SD = 5.15). The level of benefit indicated on the RBS-R+ was comparable to the level of the problem indicated by the RBS-R (t(65) = 1.59, *p* = 0.117). Participants endorsed more behaviors on the Stereotyped (t(65) = 2.01, *p* = 0.049), Compulsive (t(65) = 3.67, *p* < 0.001), and Sameness Behavior (t(64) = 2.11, *p* = 0.039) subdomains on the RBS-R+ than the RBS-R (see Table 3). The number of behaviors endorsed did not differ statistically for Self-Injurious (t(65) = 1.18, *p* = 0.242), Ritualistic (t(65) = 1.02, *p* = 0.311), or Restricted Behavior (t(64) = 1.59, *p* = 0.117) subdomains.

Also shown in Table 3, overall ratings of problem and benefit levels were comparable (t(65) = 1.59, *p* = 0.117, *d* = 0.20). Despite comparable items endorsed on each form, for Stereotyped (t(65) = 4.57, *p* < 0.001), Compulsive (t(65) = 2.44, *p* = 0.018), Ritualistic Behavior (t(65) = 2.27, *p* = 0.026), and Restricted Behavior (t(63) = 3.23, *p* = 0.002) levels of benefit were higher than the levels of problem rated. Lower benefit ratings on the Self-Injurious subdomain (t(64) = −2.61, *p* = 0.011) were consistent, with fewer of these items being endorsed on the RBS-R+.

### 3.2. Order Effect of the RBS-R and RBS-R+

There was no difference in the number of behaviors endorsed on the RBS-R (*M =* 19.06, SD = 9.67) and RBS-R+ (*M =* 19.09, SD = 8.62; Table 4) when participants completed the RBS-R+ first (t(33) = 0.03, *p* = 0.975). When the RBS-R was completed first, more items were endorsed on the RBS-R (*M* = 23.00, SD = 10.04) than the RBS-R+ (*M* = 17.19, SD = 9.89; Table 4); however, taking into account the items endorsed as present but with no benefit, totals were the same (*M* = 23.38, SD = 10.95, t(31) = 0.82; *p* = 0.42).

**Figure 1:**
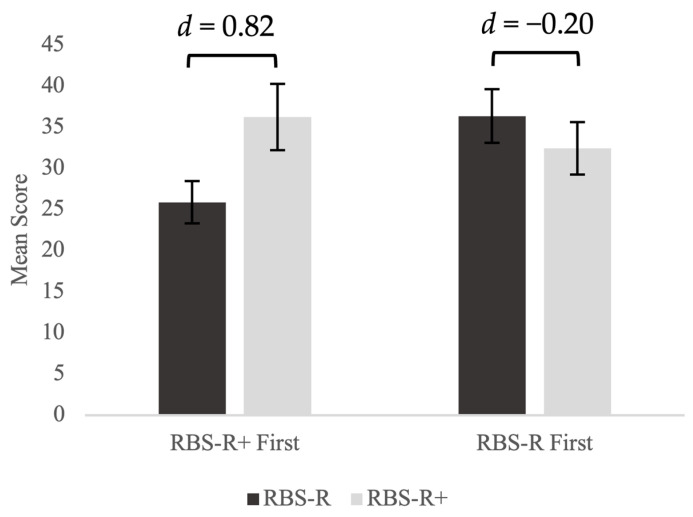
Effects of order of administration of levels of perceived benefit (RBS-R+) and problem (RBS-R).

As shown in Figure 1, when the RBS-R+ was presented first, benefit ratings on the RBS-R+ were significantly higher than problem ratings on the RBS-R (t(33) = 4.77, *p* < 0.001). This difference remained significant even after controlling for the difference in number of items endorsed (F(1,63) = 11.89, *p* = 0.001). On the other hand, there was no statistically significant difference between the total scores of the RBS-R and the RBS-R+ for participants who completed the RBS-R first (t(31) = −1.15, *p* = 0.259). Comparing the two groups, those who completed the RBS-R+ first had significantly lower ratings of problems on the RBS-R (t(64) = −2.22, *p* = 0.016) than those who completed the RBS-R first. The level of benefit was similar across administration orders (t(64) = −0.72, *p* = 0.237).

### 3.3. Relationship between Participant Characteristics and RRB Perception

*Age.* There was no relationship between age and any RBS-R or RBS-R+ scores (*r* = −0.035 to 0.023).

*Gender Identity.* The number of behaviors endorsed as problems on the RBS-R was associated with gender identity (F(2,63) = 3.14, *p* = 0.05, η^2^ = 0.09; Table 5). Post-hoc comparisons indicated that the nonbinary group endorsed significantly more RRBs than males (*p* = 0.04, *d* = −0.94). Numbers of behaviors endorsed on the RBS-R+ were comparable across the three groups (F(2,63) = 0.21, *p* = 0.812, η^2^ = 0.007).

The level of problems endorsed was not associated with gender identity (F(2,63) = 2.38, *p* = 0.101, η^2^ = 0.07), though the nonbinary group’s RBS-R mean was higher than that in females (*d* = −0.63) and males (*d* = −0.73; see Table 5). The three groups were comparable in their response to the RBS-R+ (F(2,63) = 0.19, *p* = 0.981, η^2^ = 0.01).

## 4. Discussion

Findings from this study underscore the complex nature of RRBs. Wording response options to pair RRBs occurring with levels of benefit or problem had some effect on the number of behaviors endorsed. Relative to the RBS-R (which only allows for the presence of behaviors to be rated as occurring with a mild, moderate, or severe problem), fewer behaviors were endorsed as occurring with benefit on the RBS-R+. Notably, on the RBS-R+, participants often selected the option of “behavior occurs and does not have a benefit” (*M* = 4.5 items). This suggests that only asking about benefits may result in missing some behaviors. Perhaps somewhat surprisingly, impact ratings were positively correlated, suggesting that participants viewed their behaviors as both beneficial and problematic. Similar levels of endorsement for both the benefits and problems highlight a complexity that is important to explore during diagnostic evaluations. Follow-up qualitative interviews with a subset of participants in this study reflected a range of both social, emotional, and other benefits and problems ([22,23]), which is consistent with earlier studies reporting that some behaviors may be viewed as adaptive or important to individuals while also having a negative impact due to being stigmatized by others [4,5,11].

Four out of six domains (i.e., Stereotyped Behavior, Compulsive Behavior, Ritualistic Behavior, Restricted Behavior) were rated as having higher levels of perceived benefit on the RBS-R+ (but still indicating some level of problem on the RBS-R). Further qualitative study of behaviors in these domains may be helpful to better understand the impacts of these behaviors on autistic adults. Stereotyped Behavior, which comprises behaviors often referred to as “stimming”, showed the greatest difference (M = 2.27), which aligns with studies noting these behaviors often serve as a way to regulate emotions but are often not viewed as socially acceptable [4,5]. The greater level of problem endorsed may be a reflection of both the stigma that adults perceive associated with these behaviors and resulting attempts to suppress behaviors, which may leave them without a way to regulate negative emotions.

On the other hand, on the RBS-R+, items on the Self-Injurious Behavior (SIB) and Sameness items were the most commonly rated as occurring but not having benefit, suggesting that these may be areas for the clinicians to inquire if the adult may desire some support or intervention. The RBS-R score was also significantly higher than the RBS-R+ score in the SIB subdomain, indicating that the behaviors in this domain are considered more problematic than beneficial. This is perhaps not surprising, as self-injurious behaviors may result in undesirable consequences, such as physical harm to varying degrees [11]. The more negative self-reported impact of SIBs highlights a need to understand more about the trigger and function of these behaviors to identify alternatives. The fact that some SIBs were endorsed as having benefits, however, suggests a need to understand what behaviors are being captured by this subdomain and how individuals view these behaviors as having benefits.

When the RBS-R and the RBS-R+ scores were compared across gender identity groups, males and females did not differ in the number of behaviors endorsed or the level of impact rated on either form. Although small sample sizes warrant caution in interpretation, it is notable that the nonbinary group endorsed more behaviors than males on the RBS-R only. Additionally, though not statistically significant, nonbinary individuals scored notably higher on the RBS-R than RBS-R+, indicating a higher level of problems than benefits endorsed as being associated with their behaviors. More research is needed to understand the intersection of autism and gender in understanding how autistic symptoms are experienced and expressed. It is possible that the internalization of stigma [24] after pervasive discrimination and victimization that nonbinary individuals experience throughout their lives [25] contributes to a more negative or problematic rating of their behaviors. It is also possible that gender nonbinary individuals truly experience a higher number of behaviors that fall under the RRB category, possibly relating to behaviors used to manage high levels of distress. More research is needed to understand the intersection of autism and gender in understanding how autistic symptoms are experienced and expressed.

## 5. Limitations

The present study was based on a modestly sized self-report sample of adults who responded to an online research study. It is notable that this sample has a higher proportion of females than might be expected based on prevalence estimates; however, more females are consistent with other self-report studies, particularly when the majority of the sample is adult-diagnosed [26]. Though the sample was limited with respect to racial and gender diversity, there was more variability with respect to education level and employment. In addition, the sample was comprised entirely of adults already diagnosed with ASD; future studies are needed to explore how framing behaviors more positively may affect the sensitivity and specificity of instruments in more diverse samples and in the context of first-time diagnosis.

Focus on the RBS-R could also be seen as a limitation, as coverage of sensory behaviors is limited to one item and does not capture the range of hyper- or hypo-sensitivities or sensory interests. It would be useful to have additional assessment and characterization (e.g., using observational or video-detection methods) [27,28] of their behaviors for comparison to self-report. However, it was felt that a comprehensive evaluation of RRBs was beyond the scope of this study, which aimed to explore how different framing of response options affected self-reported RRBs.

Finally, it is important to exercise caution when comparing the impact ratings. Although the scales were designed to be similar (i.e., mild, moderate, or significant benefit vs. mild, moderate, or severe problem), the scales were not identical (i.e., RBS-R+ included “behavior present but no benefit”), which may affect the direct comparison. Moreover, the repetitive nature of completing the same items twice may have affected ratings. Although the order of version presentation did not have a meaningful impact on the number of behaviors endorsed as occurring, impact ratings on the RBS-R varied based on administration order. Lower levels of negative impact were endorsed when the RBS-R+ was administered first. Impact ratings on the RBS-R+ reflected similar levels of benefit across order administrations, suggesting that order affected only the RBS-R. One possible interpretation may be that asking participants to first consider benefits associated with behaviors may prompt them to perceive their behaviors in a less negative light (i.e., thereby rating fewer problems associated with behaviors when presented with the RBS-R). This may be important to consider when developing new instruments that aim to capture both the positive and negative impacts of these behaviors.

## 6. Conclusions

Behaviors falling within the diagnostic category of RRBs are often viewed as causing impairment, and self-report measures designed to assess these behaviors often pair occurrence with negative impact. The present study demonstrates, however, that providing both negative and positive response options leads adults to endorse relatively similar numbers of behaviors. However, to ensure that the behaviors are not missed, it may be important to provide an option that allows for the endorsement of presence without benefit, as some clinically relevant behaviors may be less likely to be endorsed if the emphasis is on positive impact (e.g., SIBs). Indeed, when given the option, autistic adults endorsed both benefits and problems associated with these behaviors. These findings further underscore that a more nuanced view of RRBs is needed, as ongoing stigmatization and focus on negative aspects of behaviors can have negative impacts on individual mental health [29,30]. While demonstration of impairment in the individual’s life is required for a medical diagnosis, future screening and diagnostic tools for adults should aim to assess both problems and benefits associated with autistic features to afford a more comprehensive understanding of individual experiences while still yielding diagnostically relevant information.

## Figures and Tables

**Table 1:** Participant Demographics and Characteristics (*N* = 66).

Total N	66
**Mean Age in years (SD)**	28.24 (9.34)
**Age at diagnosis**	20.61 (SD 12.24)
Diagnosed before 18, *N* (%)	25 (37.9)
**Gender, *N* (%)**	
Female	40 (60.6)
Male	15 (22.7)
Non-binary	11 (16.7)
**Race, *N* (%)**	
White	51 (77.3)
Other *	14 (21.2)
Declined to answer	1 (1.5)
**Ethnicity, *N* (%)**	
Hispanic or Latino	9 (13.6)
**Education, *N* (%)**	
Graduate/professional degree	10 (15.2)
Baccalaureate degree	19 (28.8)
Some college/Associate degree	32 (48.5)
High school graduate	5 (7.6)
**Employed, *N* (%)**	32 (48.5)
**SRS, N (%)**	65 (98.4)
T-Score Mean (SD)	70.65 (9.96)
T-Score ≥ 60 (Mild to Severe range) *N* (%)	55 (83.3)

SD = standard deviation. * Includes Asian, Black, Native American, Native Hawaiian or Pacific Islander, and Other.

**Table 2:** Pearson Correlations between RBS-R and RBS-R+ scores.

	Total Score	Stereotyped	Self-Injurious	Compulsive	Ritualistic	Sameness	Restricted
RBS-R Standard Scoring	0.637	0.516	0.600	0.625	0.556	0.643	0.585
RBS-R items endorsed	0.746	0.767	0.621	0.739	0.669	0.568	0.778

All correlations are significant (*p* < 0.001); numbers reflect *r* between RBS-R problem and benefit scores.

**Table 3:** RBS-R and RBS-R+ scores and patterns of endorsement.

***Behaviors endorsed***	**RBS-R**	**RBS-R+**	
	*M (SD)* *occurs + problem*	*M (SD)* *occurs + benefit*	*M (SD)* *no benefit*
Total **	20.97 (9.97)	18.17 (9.23)	4.50 (5.15)
Stereotyped *	3.41 (1.91)	3.45 (1.92)	0.22 (0.63)
**Self-injurious**	3.08 (2.20)	2.08 (2.14)	1.09 (1.79)
Compulsive *	3.68 (2.34)	3.62 (2.29)	0.58 (1.16)
Ritualistic *	2.92 (1.90)	2.68 (1.86)	0.39 (0.80)
**Sameness**	5.74 (2.97)	4.29 (2.86)	1.83 (2.05)
Restricted *	2.26 (1.27)	2.00 (1.25)	0.38 (0.84)
***Impact ratings***	**RBS-R**	**RBS-R+**	
	*M (SD)* Level of problem	*M (SD)* Level of benefit	
**Total**	30.95 (19.72)	34.39 (21.36)	
Stereotyped **	4.55 (3.01)	6.82 (4.66)	
Self-injurious *	5.20 (4.63)	3.71 (4.27)	
Compulsive *	5.21 (4.21)	6.44 (5.07)	
Ritualistic *	4.18 (3.40)	5.20 (4.17)	
**Sameness**	8.69 (6.29)	7.97 (6.08)	
Restricted *	3.31 (2.63)	4.38 (3.18)	

* *p* < 0.05; ** *p* = 0.001 between RBS-R and RBS-R+.

**Table 4:** Order effect of RBS-R and RBS-R+.

***Behaviors endorsed***		**RBS-R**	**RBS-R+**
RBS-R+ First (*N* = 34)	*M* (*SD*)	19.06 (9.67)	19.09 (8.62)
RBS-R First (*N* = 32)	*M* (*SD*)	23.00 (10.03)	17.19 (9.88) *
***Level of impact***		**RBS-R**	**RBS-R+**
RBS-R+ First (*N* = 34)	*M* (*SD*)	25.88 (14.85) ^a^	36.24 (19.00) **
RBS-R First (*N* = 32)	*M* (*SD*)	36.34 (22.86) ^a^	32.44 (23.76)

* *p* < 0.05 ** *p* < 0.001 form effect (i.e., scores on RBS-R differ from RBS-R+ for that administration order); ^a^ *p* < 0.05 order effect (i.e., scores related to which form was administered first).

**Table 5:** Difference in RBS-R scores across Gender Identity.

	*Behaviors Endorsed*			*Impact Ratings*		
	***Male*** **(N = 15)**	***Female*** **(N = 40)**	***Non-Binary*** **(*N* = 11)**	***Male*** **(*N* = 15)**	***Female*** **(*N* = 40)**	***Non-Binary*** **(*N* = 11)**
**RBS-R** *M(SD)*	17.13 (11.17) ^a^	20.83 (9.32)	26.73 (8.63) ^a^	25.07 (23.74)	30.25 (17.14)	41.55 (20.39)
**RBS-R+** *M(SD)*	17.40 (8.99)	18.03 (9.57)	19.73 (8.90)	33.80 (24.74)	34.32 (21.19)	35.45 (18.88)

^a^ *p* < 0.05 across indicated gender categories.

## Data Availability

The data presented in this study are not available due to privacy.
